# The PreDoc Program: Pipeline Healthcare Apprenticeship Program through the Lens of a Neurologist

**DOI:** 10.15694/mep.2019.000063.1

**Published:** 2019-03-20

**Authors:** Rachel Marie E. Salas, Alyssa A. Gamaldo, Roy E. Strowd, Seulah Choi, Laurence T. Hou, John Shatzer, Keri Bischoff, Charlene E. Gamaldo

**Affiliations:** 1 Johns Hopkins Medicine; 2 The Pennsylvannia State University; 3 Wake Forest School of Medicine; 4 Johns Hopkins School of Education; 5 Keri Bischoff Coaching

**Keywords:** all education, premedical, mentorship, coaching, apprenticeship, healthcare, neurology, medicine, students, college

## Abstract

This article was migrated. The article was marked as recommended.

**Objective:** The PreDoc program is a longitudinal apprenticeship aimed at increasing college student interest in pursuing a healthcare career. This program offers the continuity of clinical, research, and educational exposure in academic medicine utilizing a career immersion approach that allows a graduated level of responsibility, experience, and leadership opportunities.

**Methods:** Students get an asynchronous/synchronous curriculum under the direction of academic physicians committed to boosting the pipeline. Training in critical career development skills including “goal setting,” professionalism, communication, and time management are provided to Pre-Docs by their senior peers and program leaders.

**Results:** Since the implementation of the PreDoc program in 2013, 28 students have enrolled in the program. Twenty-three students completed the survey; 100% ranked the program quality as good/excellent. Students reported more interest in academic medicine (n=19, 83%), neurology (n=18, 78%), and sleep medicine (n=18, 78%). A majority of the students reported that they were extremely likely to pursue a medical career (n=20, 87%). All students have completed or are in the process of completing at least one scholarly product.

**Conclusions:** The PreDoc program has been successful in promoting college student scholarly productivity in healthcare and in garnering student interest in academic medicine, particularly in neurology.

## Introduction

Of the 50,000+ self-identified “premed” students applying to medical school, 90% of those who eventually earn an acceptance report “working” with a physician or other healthcare professional before medical school matriculation (AAMC PMQ 2014-2017). However, when “experiential” opportunities are offered to college students, they are typically unstructured and often labeled as volunteering or shadowing experiences. These opportunities are frequently intermittent and incongruous medical exposures, in varied settings, and with inconsistent roles/expectations. Further, they are typically without standardized demonstration of progress, performance, or productivity ( Kitsis and Goldsammler 2013). While the Prematriculation Questionnaire specifically lists whether students participated in a “laboratory research apprenticeship for college students,” it does not designate other options such as apprenticeships that expose students to healthcare beyond research (AAMC PMQ 2017).

Apprenticeships allow individuals to develop a professional identity through socialization, mentorship, and experiential learning opportunities. Although healthcare has long embraced the apprenticeship paradigm, the true impact of these apprenticeships is unclear due to limitations in program uniformity or formal assessment of the short or long-term professional development outcomes for the learner. Early career exposure and interactions with positive role models within the profession, is pivotal in increasing the pool of pipeline talent into a particular field ( Allen-Ramdial and Campbell 2014; Rosenthal et al., 2013; Uppal et al., 2016; Danner et al., 2017). Timely, positive experiences with physicians also may help proactively confront concerns related to increasing reports of health professional burnout and the ongoing push to identify methods to achieve personalized work-life balance strategies. Thus, methodological and outcome studies to assess efficacious career immersion models aimed at boosting the health pipeline are needed.

There were three primary objectives of this pilot study: (1) to characterize the goals and objectives of the Pre-Doc program, (2) to define the steps and methods for implementing the Pre-Doc program, and (3) to define and measure the Pre-Doc program efficacy outcomes. Program efficacy was assessed based on the following three outcomes: (1) students’ satisfaction with the PreDoc program, (2) students’ interest and likelihood of pursuing career in heathcare, and (3) students’ scholarly productivity and overall program academic engagement.

## Methods

This study incorporated neurology preceptors and college students enrolled at Johns Hopkins University (JHU) from 2013-2017. The institutional review board approved this study and students signed a consent form.

### Objective 1. Defining the PreDoc Mission: Developing Program Statement, Goals and Objectives

The mission of the PreDoc program is to provide a modern apprenticeship that allows the apprentice (i.e., college students) to engage in the development of critical skills necessary to be successful in healthcare. The apprenticeship incorporates an organizational structure of a traditional medical residency program (i.e., internship, residency, and chief levels) to provide college students with an accelerated exposure into the medical training paradigm, and to gain some familiarity with the growing focus on team-based delivery of healthcare that involves various stakeholders (patients, clinicians, trainees, students, administrators, ancillary health providers, family members, etc.). As they progress in the program, the PreDocs receive critical professional development skills training (e.g., resiliency skills, team building, communication skills, inclusiveness and unconscious bias awareness) inarguably viewed as essential in demonstrating leadership potential. Although the program has five formal and established goals, it also offers versatility in both the educational exposure and project opportunities. These five goals of the PreDoc program are: (1) To execute an effective program using a novel, efficient curriculum that fosters development of the professional skills necessary for being a successful in healthcare; (2) To execute an effective program that cultivates an educational environment that facilitates excitement and innovation about healthcare; (3) To execute an effective program that cultivates an educational environment that promotes successful scholarly activity; (4) To execute an effective program that fosters an inclusive environment that promotes diversity; (5) To expose premedical students to interprofessional collaborative practice in healthcare.

### Objective 2. Program Design and Implementation

The PreDoc program is a credited course, thereby ensuring students are accountable. Each year, the program takes two to five students (i.e., interns) into the Academic Medicine Preview (AMP) as the initial step (See Supplemental File (SF): “Figure 1 PreDoc Overview”, Part A). Prospective students first apply for a position by completing an application and interview. Chiefs (senior PreDoc students), under the guidance of the director(s), review the applications, complete the interviews, and offer the positions to selected students. Once selected, students set learner goals ( Strowd et al., 2016) for the semester, discuss their interests and goals with the director, and meet with the chiefs for an orientation to discuss expectations, roles and responsibilities. A program curriculum (SF: “Table 1 Training Opportunities”) details the evolution of the required and optional learning opportunities in the program and (SF: “Table 2 Student Activity Tracker Examples and Narratives”) gives examples and narratives of the experiential components of the program. During the AMP semester, enrolled interns are given “mini-projects” in clinical, research, or education topics to provide students with a breadth of opportunities somewhat analogous to a medical intern. For example, a student (under the guidance of the preceptor) may develop a patient-geared educational document on narcolepsy. Each “mini-project” has a deadline with some guidance but intentionally without too many instructional specifics to encourage new ideas and perspectives (SF: “Table 2 Student Activity Tracker Examples and Narratives”). Students are monitored on accountability, quality of work, innovation, and communication. Many of these “mini-projects” and activities serve as opportunities for scholarly products or dissemination (SF: “Figure 1 PreDoc Overview”, Part B). For example, the finalized patient brochure could be handed out in clinic or as a “smart set” that could be incorporated into the electronic medical record.

It is the intention that most of the interns participating in the Academic Medicine Preview class will be selected to participate in the PreDoc program (similar to interns advancing to residents). However, this is not a guarantee. Interns must be engaged and invested in the program to be selected as PreDocs, be reliable and demonstrate an “in it to win it” attitude to be part of the team. In this way, students are more accountable and committed to the program. To personalize the opportunities to the learner interests, students meet with the director to determine interests and focus areas (analogous to a resident going into a specialty). For example, the director may ask the PreDoc to reflect on their time in the AMP and recall the “mini-projects” engaged in over the last semester to identify particular interests (SF: “Table 2 Student Activity Tracker Examples and Narratives”). There are also opportunities to recognize interests not previously exposed. The director then identifies a main project to match the PreDoc’s interest with the scholarly opportunities that are available.

### Objective 3. Measuring Program Success

Program effectiveness was assessed by number of scholarship products (e.g., manuscript), activity tracking, reflections, and satisfaction. An anonymous, online internally developed survey was also administered via Qualtrics at the conclusion of the program to assess student demographics, students’ satisfaction with PreDoc Program, and PreDoc program influencing students’ interest and likelihood of pursuing a career in healthcare. Data analysis was blinded and not performed by the directors. The number and percentage of the potential responses to each of the survey items were calculated. To examine demographic differences (e.g., group, race, and sex) across the survey items, Fisher’s exact tests were run due to the small sample size. A *p*-value of < 0.05 was considered statistically significant. All students enrolled into the AMP and PreDoc Program were eligible for inclusion in the study.

## Results/Analysis

Twenty-eight students participated in the PreDoc program since 2013 (SF: “Table 3 Participant Characteristics”). Most students were program alumni (PreDoc post-graduates), but the student population also included current PreDocs and interns (AMP Students). At the conclusion of the program, most students completed at least one “mini-project”, with the rest completing their project in the upcoming year. Moreover, 30% of alumni completed (i.e., published) three or more products (SF:“Figure 1 PreDoc Overview”, Part B).). Following the PreDoc program, most of the alumni were already in medical school or have been accepted into medical school. The rest of the alumni pursued a master’s degree or a science profession (i.e., Computer Science, Research Analyst) and were in a gap year with plans to enter a health related field (i.e., medicine, medical scientist, veterinarian). Five (all alumni) of 28 students did not complete the surveys regarding the satisfaction with the PreDoc program.

### Students’ Satisfaction with PreDoc Program

All of the students reported the overall quality was good/excellent (SF: “Table 4 Program Influence”). Most of the students reported that they (a) learned a great deal (65%); (b) the expectations for the program were the right amount (70%); (c) the program’s assignments were very/extremely useful (57%); (d) the director’s feedback was very/extremely useful (64%); (e) the director exhibited concern about them (100%); (f) the program was very good/excellent in assisting them in deciding on a medical career (78%); (g) would recommend the program to a friend (87%). Compared to current and intern students, the alumni students were more likely to report learning a great deal from the program ( *p*<.01), feeling the director’s feedback was extremely useful ( *p*<.01), and feeling the program was useful in deciding on a medical career ( *p*<.05). No gender and race/ethnic group differences were observed. Narrative comments were also collected (SF: “Table 3 Participant Characteristics”).

### PreDoc Program Influencing Students’ Interest and Likelihood of Pursuing Career in Medical Fields

As a result of the program, most of the students reported that they were more interested in academic medicine (83%), neurology (78%), and sleep medicine (78%) (SF: “Table 5 Student Satisfaction”). Although most of the students reported that they were extremely likely to pursue a medical career (87%), a high frequency of students reported that they were somewhat likely to pursue an academic medical career (48%). Although a larger proportion of alumni students reported much more interest in academic medicine as a result of the program ( *p*<.01), a larger proportion of the interns reported an extremely likely plan to pursue an academic medical career ( *p*<.05). Latino/Hispanic and Black students were more likely than East/Southeast Asian and Non-Hispanic White students to report uncertainty or not having an interest in pursuing a career in medicine (p<.01). No gender differences were observed.

## Discussion

Most colleges have premedical advising programs that are typically housed at the college campus and are not typically affiliated with a particular medical department/specialty. As a result, most students rarely have the opportunity to engage in a sustained and formal longitudinal apprenticeship with a clinician. Providing college students an opportunity to be exposed to the multi-faceted aspects of healthcare (e.g., clinical, research, administrative, etc.) using neurology as the learning environment is both feasible and effective. Here, students are introduced to various aspects of healthcare without promoting neurology explicitly but instead offering neurology as the specialty avenue to gain exposure to healthcare.

The design of the program is geared towards today’s learners. The current generation values opportunities for personal development to help understand and maximize their individual strengths ( Gallup Business Journal 2016). Students are motivated by authentic, approachable leaders and academic coaches who help them learn how they can uniquely be their best through consistent communication and feedback. Creating a shared mission with their preceptor and professional peers gives meaning to their work, making an impact on their success (Harvard Business Review 2015) and creating an educational alliance. Thus, the goal here was an apprenticeship with the directors and key preceptors to serve more in a professional “coaching” role to help students figure out their own path, medical interest, and health professional identity. The directors intentionally did not promote the program as a mentorship program. Utilizing the CliftonStrengths® workshop ( Gallup Business Journal 2016), the program has evolved to develop students as individuals to better connect them to the healthcare field. This longitudinal apprenticeship program model also enables students to gain an early appreciation for the value of cohesive team-based efforts that encompasses principles of professionalism, inclusion, and diversity as formidable skills to achieve higher productivity and success in healthcare. This promotion of interprofessional collaboration is beneficial to their future careers in healthcare, no matter the specialty. Further, in an era of immense time constraints and burnout as early as medical school up through the faculty level, novel ways to develop a win-win relationship are of necessity. This PreDoc paradigm not only provides an environment to develop stronger relationships, but also balances the efforts on both faculty and students allowing for scholarly productivity and improved time management (e.g., chiefs doing training and providing guidance for tasks).

The directors of the PreDoc program were initially junior faculty members when this program was created and were also the clerkship co-directors in neurology. This connection was important to provide opportunities for the PreDocs to engage with medical students and house staff. While there is no full-time equivalent (FTE) support for the directors through the PreDoc program, productivity benefits were considered of value to sustain involvement. The program is currently composed of four key faculty members (two co-directors, one research PreDoc Preceptor, and one clinical PreDoc Preceptor). Further, the co-directors now have other leadership roles (e.g., Director of Interprofessional Education and Collaborative Practice and Vice Chair for Faculty development in the department) which provides more opportunities for PreDocs. While PreDocs are exposed to other faculty and healthcare members, the core is kept small intentionally to promote a tight leadership unit.

Exposing PreDocs to a diverse group of faculty and healthcare leaders has been a strategic goal for this program, especially since medicine continues to address a widening gap in the number of under-represented in medicine (UIM) physicians. The two PreDoc co-directors are UIM female faculty. Allowing students to work closely with faculty who represent diverse backgrounds affords the opportunity for students to implicitly and explicitly envision themselves or others (regardless of background) in healthcare roles and even rising into leadership roles. While 83% of students were more interested in academic medicine and 78% were more interested in a career in neurology, Latino/Hispanic and Black students were more likely to report uncertainty or not having an interest in pursuing a career in medicine. This may be skewed as many of these students are either interns or current PreDocs and are still figuring out their interest. Future investigation regarding the unique role that apprentice and preceptor demographics plays in the program outcome metrics are critical for understanding optimal models that maybe unique for enhancing the pipeline for UIM talent. While this is only pilot data and the program is still in the early stages, one cannot argue the premise of introducing healthcare to college students in this thoughtfully created environment may be a novel way to strengthen the healthcare pipeline.

## Conclusion

The PreDoc Program offers an effective, structured academic medicine apprenticeship that prepares students for a career in healthcare with increased interest in academic medicine. The program incorporates new approaches proven to be effective in the business arena for career development that integrates a diverse perspective and engages a vertical horizontal team-based approach modeled after a medical residency. The PreDoc program also provides an earlier opportunity and platform for students to develop an appreciation for scholarly productivity, career coaching and professional development.

## Take Home Messages

• The PreDoc program is a longitudinal apprenticeship aimed at increasing college student interest in pursuing a healthcare career.
• Training in critical career development skills including goal setting, professionalism, communication, and time management are provided to Pre-Docs by their senior peers and program leaders.
• Students reported more interest in academic medicine, neurology, and sleep medicine.
• All students have completed or are in the process of completing at least one scholarly product.
• The PreDoc program has been successful in promoting college student scholarly productivity in healthcare.

## Notes On Contributors

Rachel Marie E. Salas, MD, MEdHP, FAAN is an Associate Professor, Neurology and Nursing at Johns Hopkins Medicine Director of Interprofessional Education and Interprofessional Collaborative Practice for the School of Medicine; Director, Neurology Clerkship Director for the School of Medicine, PreDoc Program for the Johns Hopkins University. ORCHID: https://orcid.org/0000-0002-3945-3336

Alyssa A. Gamaldo, PhD is an Assistant Professor, Human Development and Family Studies at the Pennsylvania State University. ORCHID: https://orcid.org/0000-0003-0072-5151

Roy E. Strowd, MD, MEdHP is an Assistant Professor, Neurology, Internal Medicine, Section on Hematology and Oncology at Wake Forest School of Medicine in Winston-Salem, North Carolina. He is Director of the Health Professions Education Institute at Wake Forest and focuses on leadership development and mentoring in pre-medical, medical, and post-graduate health professions education. ORCHID: https://orcid.org/0000-0001-6651-5267

Seulah Choi, BA is a medical student at Icahn School of Medicine at Mount Sinai, New York (MSSM).

Laurence T. Hou, BA is an M. D. Candidate - The Johns Hopkins School of Medicine. ORCHID: https://orcid.org/0000-0001-7066-7378

John Shatzer, PhD Associate Professor at Johns Hopkins School of Education. ORCHID: https://orcid.org/0000-0003-4098-9143

Keri Bischoff, MAED. As a licensed teacher, educational leader, and business owner, Keri uses her knowledge of training, development, and organizational strategy to contribute to the growth of powerful teams. She holds an undergraduate degree in Business Management and Organizational Behavior, and a Master of Arts degree in Education. She is certified as a Bridgeworks Generational trainer.

Charlene E. Gamaldo, MD, FAAN, FAASM is an Associate Professor, Neurology, Psychiatry, Anesthesia/Critical Care, Nursing and Public Health Vice Chair, Faculty Development - Neurology Department and Medical Director

JH Center for Sleep. ORCHID: https://orcid.org/0000-0002-9386-9666

## Declarations

The author has declared that there are no conflicts of interest.

## Ethics Statement

This study was approved by Johns Hopkins IRB #HIRB05023.

## External Funding

This article has not had any External Funding
