# Supplementary material for: The PreDoc Program: Pipeline Healthcare Apprenticeship Program through the Lens of a Neurologist
Source: MedEdPublish (2016). 2019 Mar 20;8:63. [Version 1] doi: 10.15694/mep.2019.000063.1 (PMC10712643; doi:10.15694/mep.2019.000063.1)
Supplement: Peer review history

# Reviewer report: Reviewer response for version 1

**DOI:** 10.21956/mep.19749.r29625

**Published:** 2019-07-12

**Authors:** Ken Masters

**Affiliations:** 1 Sultan Qaboos University

This review has been migrated. The reviewer awarded 4 stars out of 5 An interesting paper describing a “PreDoc” program which is a longitudinal apprenticeship aimed at increasing college student interest in pursuing a healthcare career. The authors lay out the central problem of inconsistent and unverified apprenticeships and other pre-exposures of medical students to the medical world prior to training. The PreDoc program aims at addressing this problem.The program is well described, and the authors have used the supplementary files to give a large amount of useful detail regarding the course, to the point that others could relatively easily take the program and adapt it to their own needs and circumstances. The evaluation allows for a coherent assessment of the impact of the program.There does, however, need to be some clarity on the enrolment method. The project is described as a Pilot, so this may not yet have been resolved. In addition, one would wish to measure these results against some sort of control group.Still, an interesting and useful program. At the very least, it gives a clear indication to those who realise that they are not suited to medicine, and allows them to change course at an early stage of their academic career.

Reviewer Expertise:

NA

No decision status is available

# Reviewer report: Reviewer response for version 1

**DOI:** 10.21956/mep.19749.r29624

**Published:** 2019-03-28

**Authors:** John Cookson

**Affiliations:** 1 University of Worcester

This review has been migrated. The reviewer awarded 4 stars out of 5 Thank you, I enjoyed reading this. Most medical schools look for some evidence of engagement with health care in the applications they receive. However the response from students can be quite variable and it has been noted that students from more affluent backgrounds are more likely to be able to organise such attachments. It is therefore helpful to see a programme that has been formally organised. Two questions however, I was not sure how students were selected and whether WP played any part. Also, who pays? The costs may be hidden but hey are still there.

Reviewer Expertise:

NA

No decision status is available
